# Supplementary material for: Morphogenesis of Fractofusus andersoni and the nature of early animal development
Source: Nat Commun. 2025 Apr 11;16:3439. doi: 10.1038/s41467-025-58605-9 (PMC11985926; doi:10.1038/s41467-025-58605-9)
Supplement: Supplementary file 1 — Supplementary information [file 41467_2025_58605_MOESM1_ESM.pdf]

Supplementary Figures:

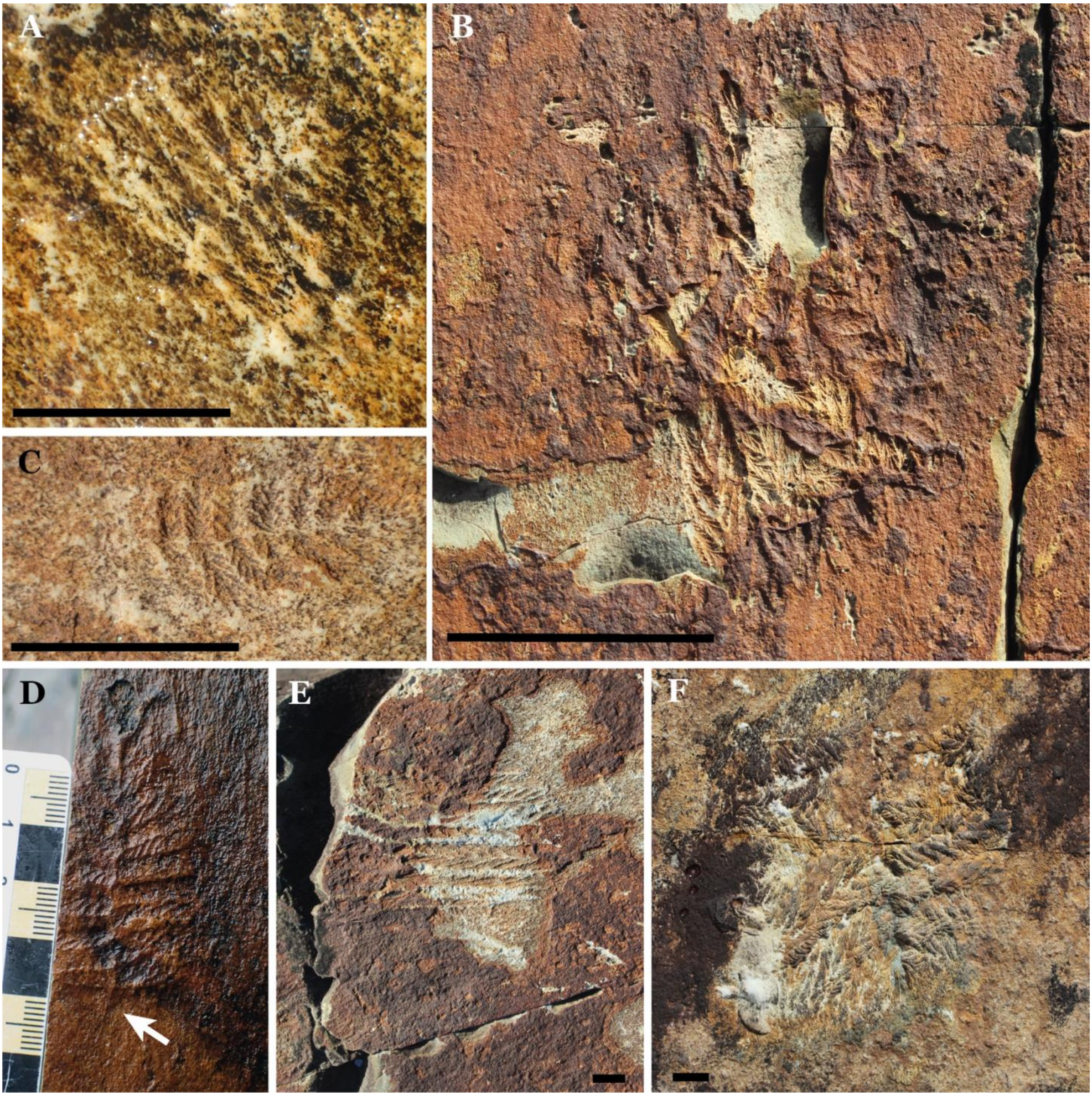

Supplementary Figure 1: Additional 'broken' field specimens. All specimens from Br5. White arrow in (D) indicates stolon continuing from midline of specimen. Scale bars in A, C, E & F = 1cm. Scale bar in B = 5cm.

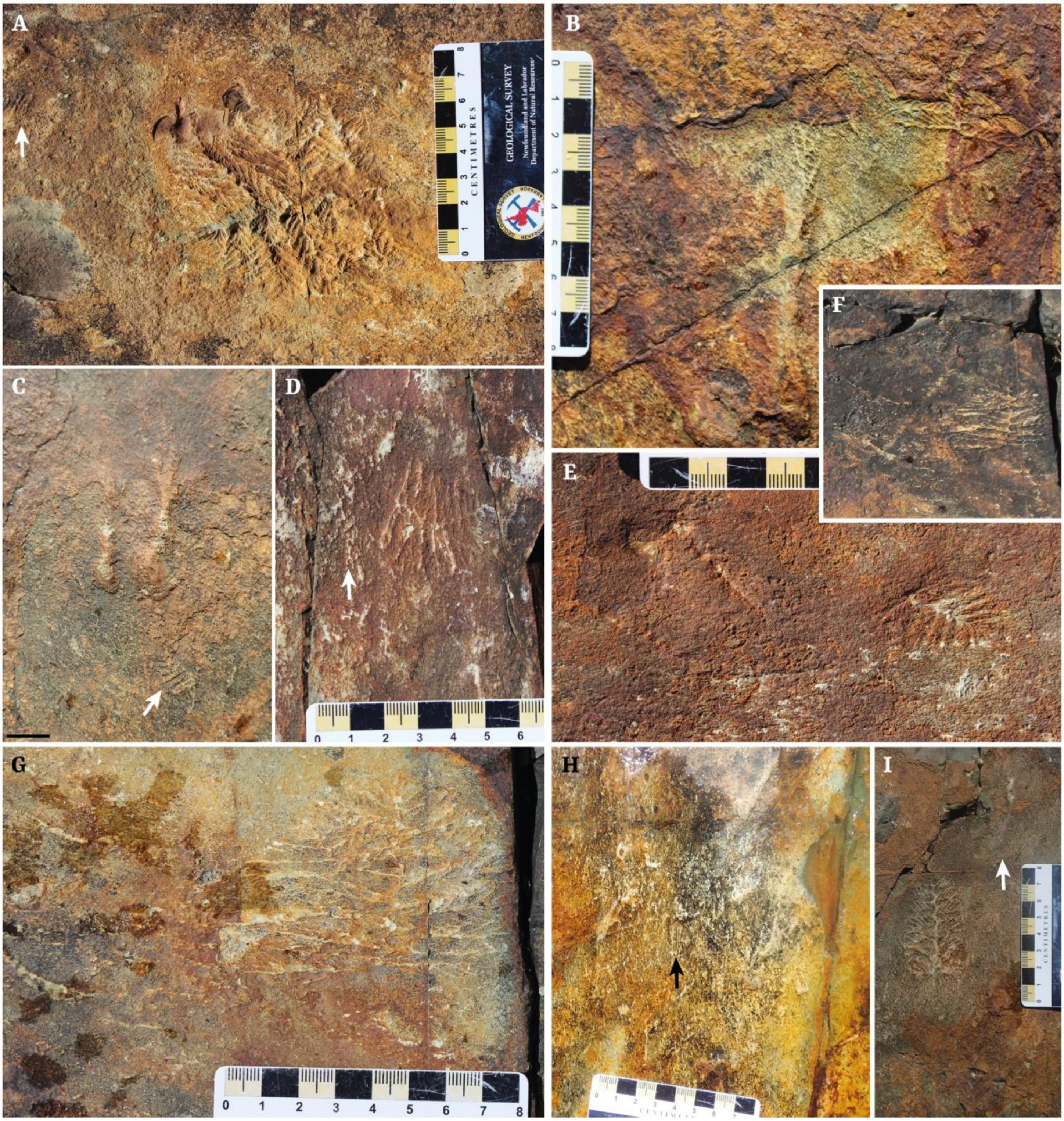

Supplementary Figure 2: Incomplete specimens from surface BR5 with contextual information. A) Two incomplete specimens (smaller specimen arrowed) preserved at different orientations. B) Incomplete specimen preserved in same orientation as felled upright fronds. C) Incomplete specimen (arrowed) preserved at orientation 129 degrees separated from upright fronds. D) Incomplete specimen preserved at orientation 46 degrees separated from upright fronds (arrowed). E) Incomplete specimen preserved at orientation 22 degrees separated from upright fronds. F) Incomplete specimen preserved at orientation 67 degrees separated from upright fronds. G) Incomplete specimen preserved at orientation 60 degrees separated from upright fronds. H) Incomplete specimen (arrowed) preserved at orientation 58 degrees separated from upright fronds. I) Incomplete specimen preserved at orientation 25 degrees separated from upright frond (arrowed). Scale bars = 1cm.

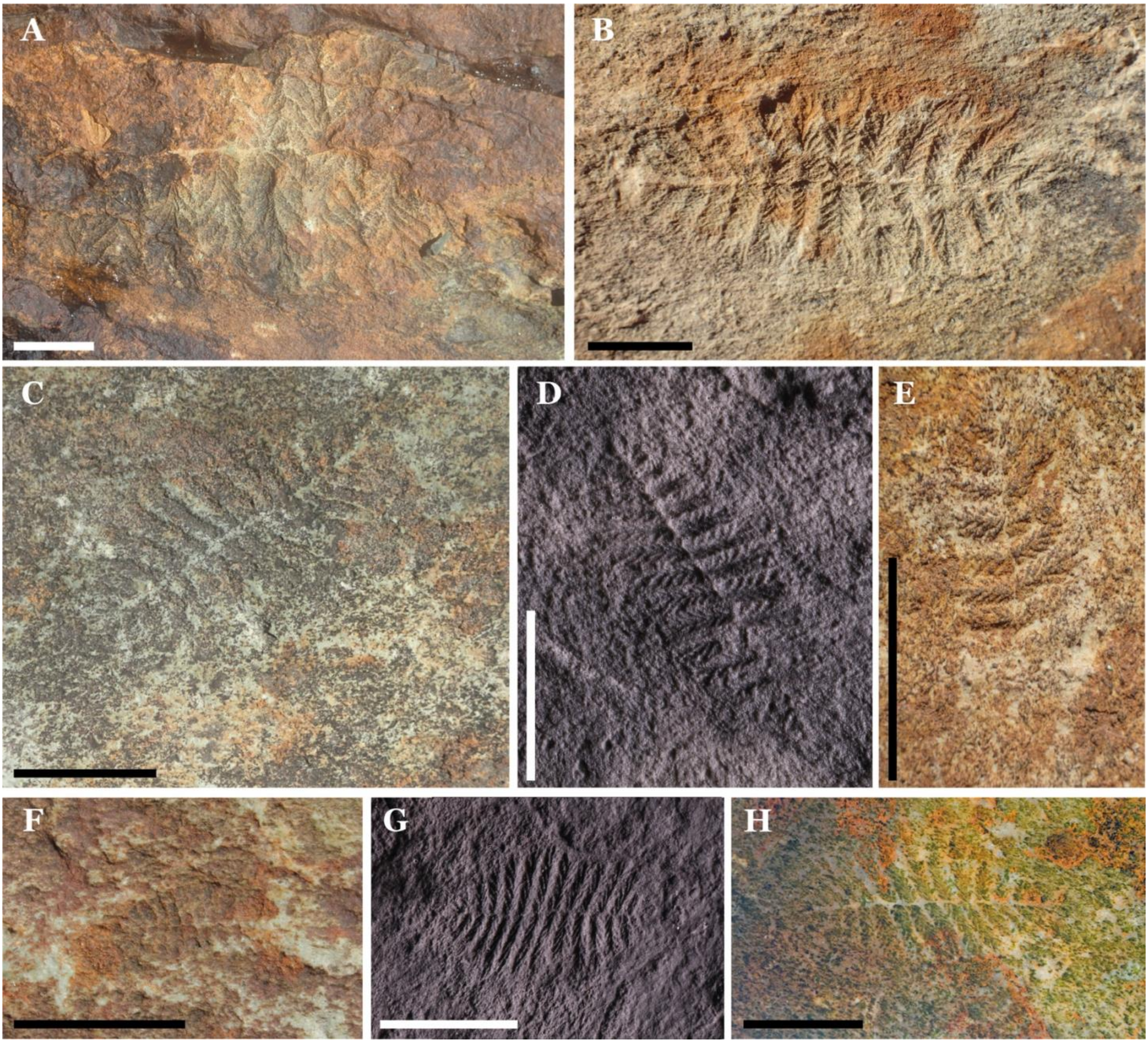

Supplementary Figure 3: Additional specimens showing a centrally located, bilateral pair of branches. Specimens A-C, E-F and H are field specimens. D) is specimen X.50400.5. G) is specimen X.50400.7. All scale bars = 1cm.

Specimen size (mm)	**9.8**	**10.2**	**14.5**	**17.0**	**24.2**	**34.7**	**67.7**
**7.8**	0.8001	0.9619	0.8462	0.3478	0.0008	0.0002	9.25E-05
**9.8**		0.6589	0.445	0.1428	7.6363E-05	5.02E-06	4.38E-06
**10.2**			0.8354	0.0876	0.0014	0.0002	0.0007
**14.5**				0.0422	0.0004	4.02E-05	7.37E-05
**17**					0.0006	5.09E-05	2.52E-04
**24.2**						0.2488	0.985
**34.7**							0.1873

Supplementary Figure 4: Wald test of estimated coefficients, comparing the slope of the outline of *F. andersoni* of different sizes from the BR5 fossil surface. Significantly different slopes are shown in green and slopes which are not statistically indistinguishable are shown in red. Specimen size (in mm) of pairwise specimen comparisons are shown in bold. Data available in Supplementary dataset 2.
