# Supplementary material for: Morphogenesis of Fractofusus andersoni and the nature of early animal development
Source: Nat Commun. 2025 Apr 11;16:3439. doi: 10.1038/s41467-025-58605-9 (PMC11985926; doi:10.1038/s41467-025-58605-9)
Supplement: Supplementary file 2 — Description of additional supplementary files [file 41467_2025_58605_MOESM2_ESM.pdf]

Description of additional supplementary files

Supplementary dataset 1: RTI files associated with specimens figured in main text Fig. 2 G-L.

Supplementary dataset 2: Excel sheet presenting all original specimen measurement data.

Supplementary dataset 3: Excel sheet comparing total specimen length with number of first order branches

Supplementary dataset 4: Excel sheet total specimen length with the maximum observed number of second order branches per first order branch

Supplementary dataset 5: Excel sheet total specimen length with the length of the central first order branch.

Supplementary dataset 6: Excel sheet total specimen length with the observed skew from a normal distribution in first order branch length across one vane of the specimen (branch length data available in Supplementary dataset 1).

Supplementary dataset 7: Excel sheet total specimen length with the maximum branch length (the central branch) and the number of first order branches observed in a given specimen.

Supplementary dataset 8: Excel sheet total specimen length with the length of the distal-most branch preserved, where tip preservation was deemed complete enough to collect these measurements.

Supplementary dataset 9: List of all specimen casts accessioned (and accession codes), including figured specimens and those which quantitative data was derived from alongside additional specimens.

Supplementary code 1: R script required to reproduce all analyses.
