# Supplementary material for: Morphogenesis of Fractofusus andersoni and the nature of early animal development
Source: Nat Commun. 2025 Apr 11;16:3439. doi: 10.1038/s41467-025-58605-9 (PMC11985926; doi:10.1038/s41467-025-58605-9)
Supplement: Supplementary file 7 — Reporting Summary [file 41467_2025_58605_MOESM7_ESM.pdf]

Corresponding author(s): Frances Dunn

Last updated by author(s): 20215 - 03 - 14

Reporting Summary

Nature Portfolio wishes to improve the reproducibility of the work that we publish. This form provides structure for consistency and transparency in reporting. For further information on Nature Portfolio policies, see our Editorial Policies and the Editorial Policy Checklist.

Statistics

For all statistical analyses, confirm that the following items are present in the figure legend, table legend, main text, or Methods section.

n/a	Confirmed	
☐	☒	The exact sample size (n) for each experimental group/condition, given as a discrete number and unit of measurement
☐	☒	A statement on whether measurements were taken from distinct samples or whether the same sample was measured repeatedly
☐	☒	The statistical test(s) used AND whether they are one- or two-sided<br>*Only common tests should be described solely by name; describe more complex techniques in the Methods section.*
☐	☒	A description of all covariates tested
☐	☒	A description of any assumptions or corrections, such as tests of normality and adjustment for multiple comparisons
☐	☒	A full description of the statistical parameters including central tendency (e.g. means) or other basic estimates (e.g. regression coefficient) AND variation (e.g. standard deviation) or associated estimates of uncertainty (e.g. confidence intervals)
☐	☒	For null hypothesis testing, the test statistic (e.g. F, t, r) with confidence intervals, effect sizes, degrees of freedom and P value noted<br>*Give P values as exact values whenever suitable.*
☒	☐	For Bayesian analysis, information on the choice of priors and Markov chain Monte Carlo settings
☒	☐	For hierarchical and complex designs, identification of the appropriate level for tests and full reporting of outcomes
☒	☐	Estimates of effect sizes (e.g. Cohen's d, Pearson's r), indicating how they were calculated

Our web collection on statistics for biologists contains articles on many of the points above.

Software and code

Policy information about availability of computer code

Data collection	All data associated with this study is released with this study. This includes a list of specimens used (with corresponding accession numbers at the Sedgwick Museum of Earth Sciences) with all measurement data and RTI files for significant specimens.
Data analysis	R (version 4.4.0) was used for data analysis and an annotated R script used to run all analyses is available with this manuscript.

For manuscripts utilizing custom algorithms or software that are central to the research but not yet described in published literature, software must be made available to editors and reviewers. We strongly encourage code deposition in a community repository (e.g. GitHub). See the Nature Portfolio guidelines for submitting code & software for further information.

Data

Policy information about availability of data

All manuscripts must include a data availability statement. This statement should provide the following information, where applicable:

- Accession codes, unique identifiers, or web links for publicly available datasets
- A description of any restrictions on data availability
- For clinical datasets or third party data, please ensure that the statement adheres to our policy

Our data availability statement highlights accession numbers of specimens, the museum they are held in and provides contact information for the relevant collections manager.

Research involving human participants, their data, or biological material

Policy information about studies with human participants or human data. See also policy information about sex, gender (identity/presentation), and sexual orientation and race, ethnicity and racism.

Reporting on sex and gender	NA
Reporting on race, ethnicity, or other socially relevant groupings	NA
Population characteristics	NA
Recruitment	NA
Ethics oversight	NA

Note that full information on the approval of the study protocol must also be provided in the manuscript.

Field-specific reporting

Please select the one below that is the best fit for your research. If you are not sure, read the appropriate sections before making your selection.

☐ Life sciences ☐ Behavioural & social sciences ☒ Ecological, evolutionary & environmental sciences

For a reference copy of the document with all sections, see nature.com/documents/nr-reporting-summary-flat.pdf

Ecological, evolutionary & environmental sciences study design

All studies must disclose on these points even when the disclosure is negative.

Study description	We assessed morphogenesis in a single population of the rangeomorph Fractofusus andersoni from the Br5 site of the Mistaken Point Ecological Reserve by comparing the number and length of individual branches between specimens.
Research sample	We used a single population of Fractofusus andersoni from one fossil site in order to reduce the likelihood of the confounding effects of ecophenotypic variation.
Sampling strategy	The best preserved specimens across the size range (~1-6cm) were selected for analysis. These specimens were photographed from casts (where moulds were made in the field, and jesmonite casts were made in the lab).
Data collection	Specimens were photographed and branch information was counted and recorded by FD.
Timing and spatial scale	NA
Data exclusions	Specimens which were not complete were excluded from our study on morphogenesis as they did not retain enough original morphological information.
Reproducibility	All specimens used will be accessioned in the Sedgwick museum, Cambridge and all data collected is available with this study.
Randomization	NA
Blinding	NA

Did the study involve field work? ☒ Yes ☐ No

Field work, collection and transport

Field conditions	East coast of Newfoundland, Canada between the months of May and September.
Location	Mistaken Point Ecological Reserve, Newfoundland, Canada. Site Br5 of Liu et al. 2016 (Palaeontology)
Access & import/export	We procured field permits required to enter the fossil sites and make silicon molds of specimens within the reserve from Mistaken Point Ecological Reserve.

Disturbance	No disturbance was caused by this study. All casting materials were cleaned up in situ, as per the terms of permit issued.

Reporting for specific materials, systems and methods

We require information from authors about some types of materials, experimental systems and methods used in many studies. Here, indicate whether each material, system or method listed is relevant to your study. If you are not sure if a list item applies to your research, read the appropriate section before selecting a response.

Materials & experimental systems

n/a	Involved in the study
☒	☐ Antibodies
☒	☐ Eukaryotic cell lines
☐	☒ Palaeontology and archaeology
☒	☐ Animals and other organisms
☒	☐ Clinical data
☒	☐ Dual use research of concern
☒	☐ Plants

Methods

n/a	Involved in the study
☒	☐ ChIP-seq
☒	☐ Flow cytometry
☒	☐ MRI-based neuroimaging

Palaeontology and Archaeology

Specimen provenance	*Provide provenance information for specimens and describe permits that were obtained for the work (including the name of the issuing authority, the date of issue, and any identifying information). Permits should encompass collection and, where applicable, export.*
Specimen deposition	The specimens have been deposited in the Sedgwick Museum, Cambridge.
Dating methods	NA

☐ Tick this box to confirm that the raw and calibrated dates are available in the paper or in Supplementary Information.

Ethics oversight	No ethical approval was required. All field access and mould making was done in accordance with permits received from the Mistaken Point Ecological Reserve.

Note that full information on the approval of the study protocol must also be provided in the manuscript.

Plants

Seed stocks	NA
Novel plant genotypes	NA
Authentication	NA
